# Functional Components of Carob Fruit: Linking the Chemical and Biological Space

**DOI:** 10.3390/ijms17111875

**Published:** 2016-11-10

**Authors:** Vlasios Goulas, Evgenios Stylos, Maria V. Chatziathanasiadou, Thomas Mavromoustakos, Andreas G. Tzakos

**Affiliations:** 1 Department of Agricultural Sciences, Biotechnology and Food Science, Cyprus University of Technology, Lemesos 3603, Cyprus; vlasios.goulas@cut.ac.cy; 2 Section of Organic Chemistry and Biochemistry, Department of Chemistry, University of Ioannina, 45110 Ioannina, Greece; evstylos@gmail.com (E.S.); m.chatziathanasiadou@gmail.com (M.V.C.); 3 Biotechnology Laboratory, Department of Biological Applications and Technologies, University of Ioannina, 45110 Ioannina, Greece; 4 Laboratory of Organic Chemistry, Department of Chemistry, National and Kapodistrian University of Athens, 11571 Athens, Greece; tmavrom@chem.uoa.gr

**Keywords:** carob, locust bean gum, fiber, polyphenols, tannins, hyperlipidemia, diabetes, cancer, diarrhea, omics

## Abstract

The contribution of natural products to the drug-discovery pipeline has been remarkable since they have served as a rich source for drug development and discovery. Natural products have adapted, during the course of evolution, optimum chemical scaffolds against a wide variety of diseases, including cancer and diabetes. Advances in high-throughput screening assays, assisted by the continuous development on the instrumentation’s capabilities and omics, have resulted in charting a large chemical and biological space of drug-like compounds, originating from natural sources. Herein, we attempt to integrate the information on the chemical composition and the associated biological impact of carob fruit in regards to human health. The beneficial and health-promoting effects of carob along with the clinical trials and the drug formulations derived from carob’s natural components are presented in this review.

## 1. Introduction

The carob tree (*Ceratonia siliqua* L.) belongs to the Leguminosae family and is widely cultivated in the Mediterranean area where it is considered an important component of vegetation for economic and environmental reasons [1]. World production is estimated at about 160,000 t/year produced from some 80,000 hectares with very variable yields depending on cultivar, region and farming practice [2]. The fruit is an indehiscent pod, elongated, compressed, straight or curved, thickened at the sutures, 10–30 cm long, 1.5–3.5 cm wide and about 1 cm thick with blunt or subacute apex. Pods are brown with a wrinkled surface and are leathery when ripe. The pulp comprises an outer leathery layer (pericarp) and softer inner region (mesocarp). Seeds occur in the pod transversally, separated by mesocarp [3].

The carob fruit contains two major parts: the pulp (90%) and the seeds (10%). The chemical composition of the pulp depends on cultivar, origin and harvesting time. Carob pulp is high (48%–56%) in total sugar content (mainly sucrose, glucose and fructose). In addition, it contains about 18% cellulose and hemicellulose. Ripe carob pods also contain a large amount of condensed tannins (16%–20% of dry weight) [4].

Carob seeds are usually exploited for the production of carob bean gum (CBG) or locust bean gum (LBG). This gum comes from the endosperm of the seed and chemically is a galactomannan. It is added as thickener, stabilizer or flavoring in food. In addition to the food industry, LBG is widely used for pharmaceutical purposes as it is linked with the inhibition of gastrointestinal diseases [5,6,7,8]. Furthermore, LBG is exploited as a carrier agent for the controlled release of drugs alone or in combination with other carrier molecules [9,10,11,12,13,14,15,16].

Recently, researchers have focused on the valorization of carob pods since they are an excellent source of bioactive compounds such as dietary fiber, polyphenols, and cyclitols and contain low amounts of fat. In addition, carob pods can be used as a cocoa substitute since they do not contain caffeine and theobromine. The whole unprocessed fruit or its by-products, such as the germ, fruit extract, kibbles without seeds and the seed peel, have also been investigated by food technologists. Different parts of carob fruits have been used as food ingredients in bakery and confectionery products [17,18], as well as in fermented and non-fermented pastas due to their health-promoting properties [19,20,21]. Furthermore, researchers have attempted to formulate carob-based milk beverages and decoctions [22,23]. Carob pods are an ideal substrate for the production of food ingredients exploiting biotechnology. In particular, they are mainly used to produce citric acid [24], lactic acid [25], mannitol [26], succinic acid [27] and ethanol [28].

In the last two decades, numerous studies have demonstrated interesting findings concerning the bioactivity of carob pulp constituents. Fiber, cyclitols, polyphenols and tannins have mainly attracted scientific attention. These groups of bioactive compounds have been linked with the health-promoting effects of carob in different therapeutic areas, including anti-cancer, anti-diabetes, anti-diarrheal and anti-hyperlipidemia [29,30,31,32,33,34]. These findings have rendered carob fruit an excellent ingredient for the development of functional food and herbal supplements. The valorization of these bioactive constituents is more attractive if we consider that they are usually discarded as LBG and simple sugars are used by the industry.

In the present review, the beneficial effects of carob fruit are demonstrated. In an attempt to review critically its health-promoting effects, the nutritional and bioactive composition is presented, followed by specific effects on human health. A special focus has been also given to clinical trials and carob’s drug formulations.

## 2. Functional Chemical Components of Carob Fruit

**Figure 1:**
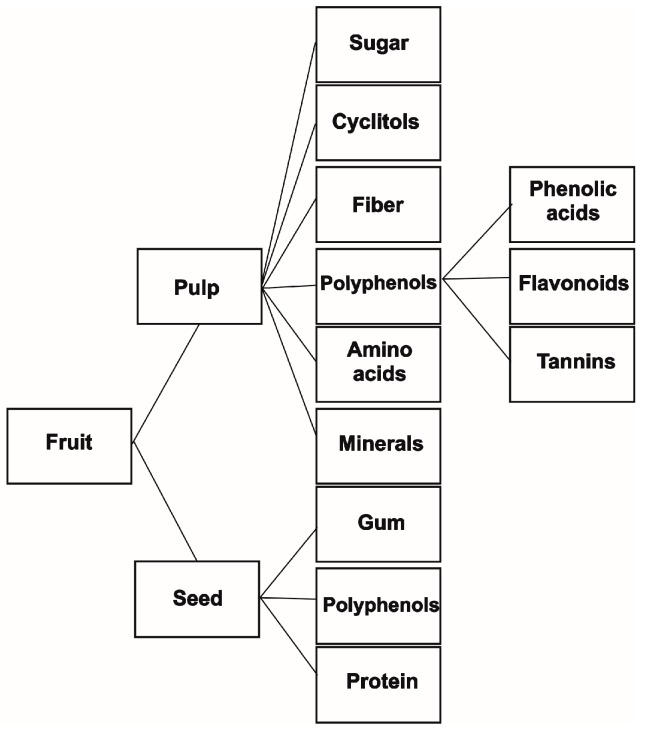
Main chemical constituents in carob pulp and seed with nutritional and health-promoting properties.

Carob fruit is a complex mixture of primary and secondary metabolites, with the presence of sugars and fibers being characteristic for these fruits, followed by a great diversity of polyphenols. Numerous minerals and amino acids are also present in carob fruits. Figure 1 summarizes the major constituents in carob pulp and seed. Here, we present the chemical components of carob fruit with bioactivity and/or economic importance.

### 2.1. Sugars

Carob fruit is known for its high sugar content that is responsible for the nutritional value of beans. Previous studies reported that total sugar content in the cultivars ranged between 40 and 55 g·100 g^−1^·d.m. [3,35,36]. In general, cultivated carob cultivars have higher sugar content than wild ones [35]. Regarding the sugar composition, sucrose is the major carbohydrate in carob bean and its concentration can be reached up to 52 g 100 g^−1^·d.m. [3,35]. Fructose and glucose with 1.8–12.5 g 100 g^−1^·d.m. and 1.8–10.2 g 100 g^−1^·d.m. concentrations, respectively, are also present in carob bean [35,36]. Carob sugars are usually extracted for the production of natural carob syrup. Furthermore, an innovative process has been patented for their recovery in order to produce carob syrup [37].

### 2.2. Cyclitols

**Figure 2:**
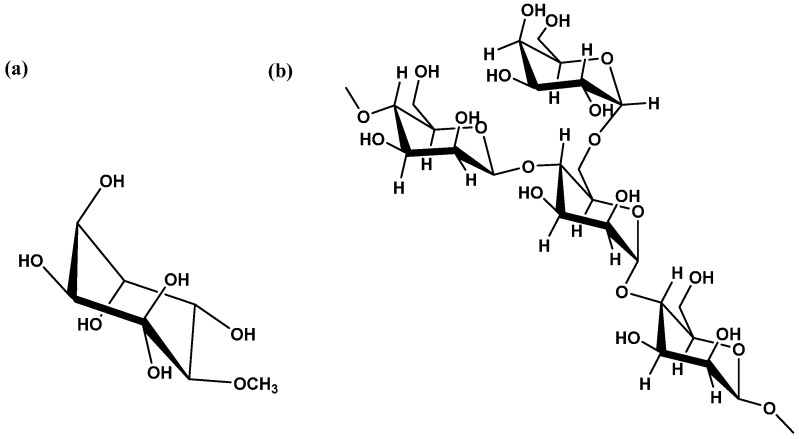
Chemical structures of (**a**) d-pinitol and (**b**) locust bean gum (LBG).

As all legumes, a set of cyclitols with multiple health benefits in carob bean has been confirmed [38]. In carob bean, the major cyclitol is d-pinitol (3-*O*-methyl-d-chiro-inositol) and its content showed great diversity (1.0–8.5 g 100 g^−1^·d.m.) (Figure 2a). The concentration of d-pinitol is influenced by genetic and environmental factors; especially the mean d-pinitol content of wild carob cultivars is higher than mean d-pinitol content of cultivated ones [35]. The presence of d-pinitol is of high importance as it can be used as a marker of carob adulteration by cocoa [39]. In addition, carob bean can be considered as an excellent reservoir of d-pinitol and its isolation procedure has been patented [40]. Recently, ultrasound-assisted extraction and supercritical fluid extraction have been proposed for the isolation of d-pinitol from carob [23,41]. Finally, myo-inositol, d-(+)-chiro-inositol, ononitol (4-*O*-methyl-myo-inositol), sequoyitol (5-*O*-methyl-myo-inositol) and bornesitol (1-*O*-methyl-myo-inositol) have been detected only at trace levels [39].

### 2.3. Fibers

Dietary fiber is a heterogeneous group of substances, commonly divided into soluble and insoluble fibers. Carob fiber is produced by water extraction of the carob pulp to remove the majority of soluble carbohydrates; total dietary fiber content usually ranged 30%–40% of carob pulp [41]. A method of making the natural carob fiber has also been patented [42]. The insoluble dietary fiber fraction consists of cellulose, hemicelluloses, lignin and insoluble polyphenols and its minimum content exceeds 70% of carob fiber. The high proportion of polyphenols present in carob fiber differentiates it from other dietary fiber sources. In general, carob fiber is considered as a predominantly insoluble and practically non-fermentable dietary fiber [43]. On the other hand, the amount of soluble dietary fiber is significantly lower (max 10 g 100 g^−1^ carob fiber) and contains simple carbohydrates as we discussed thoroughly in the previous section. Finally, carob fiber has a great effect on dough rheology when it is used as an ingredient in bakery products [44,45].

### 2.4. Gum

LBG is a white to creamy white powder obtained from the seed endosperm of the fruit pod of the carob tree. It is comprised of a high molecular weight polysaccharide composed of galactomannan and its concentration can be reached up to 85% of carob seed. Galactose and mannose are the two components of LBG. In particular, locust bean galactomannan consists of a linear chain of (1→4)-linked α-d-mannopyranosyl units with (1→6)-linked α-d-galactopyranosyl residues as side chains (Figure 2b). The galactose to mannose ratio in LBG has been calculated between 1:3.1–1:3.9 and mannose and galactose content has been reported as 77%–78% and 21%–23%, respectively. The distribution of d-galactosyl residues or side chains along the mannose backbone chain can be random, blockwise and ordered. The molecular size and structure of galactomannans are of great importance, as they greatly affect the functional properties of CBG [46,47]. Functional properties of LBG such as solubility, rheology, viscosity, hydration rate, synergistic gel formation and water adsorption have been studied [48].

Finally, many patents describe the novel use of LBG in food products such as jelly foods, baby food etc. [49,50]. LBG is also exploited for the development of novel barriers to improve organoleptic characteristics of food products [51].

### 2.5. Polyphenols

Polyphenols constitute one of the most common and widespread groups of substances in plants. Several thousand plant polyphenols are known, encompassing a wide variety of molecules consisting of one or more aromatic rings with variable degrees of hydroxylation, methoxylation and glycosylation [52]. The main categories of phenolic compounds found in carob fruit are phenolic acids, gallotannins and flavonoids. The concentration of polyphenols in carob fruits depends strongly on genetic, environmental and extraction methods and ranges between 45–5376 mg gallic acid equivalents per 100 g [53,54,55]. In carob fruits, phenolic compounds are found as free, as bound or as soluble conjugated forms; Dubravka et al. reported that the majority of carob phenolics are covalently bound to the dietary fibers [56]. In addition, carob germ and carob seed are rich sources of phenolic compounds [1]. Polyphenolic composition of different parts of carob is presented in Table 1. Carob polyphenols have attracted scientific interest; thus many extraction methods have been proposed to recover polyphenols from carob [55,57,58]. In addition, a patent towards the extraction and purification of phenolic compounds has been registered [59].

Phenolics, subdivided into benzoic and cinnamic acids, are the most abundant class of polyphenols in carob fruits. Indeed, gallic acid and its derivatives such as methyl gallate comprise the majority of phenolic acids [53,61]. Carob fruit is one of the richest source of gallic acid as its content has been estimated between 23.7 mg 100 g^−1^ and 164.7 mg 100 g^−1^ [53,61,63]. According to the Phenol-Explorer database, only chestnut and cloves had higher gallic acid content than carob fruits. Syringic acid, 4-hydrobenzoic acid, and gentisic acid are also benzoic acids that are found in carob fruit [53,57,62]. The concentration of cinnamic acids in carob fruit is relatively low; cinnamic acid, coumaric acid, ferulic acid and chlorogenic acid have been identified in carob fruit extracts [57,62,64].

Flavonoids represent the most diverse group of phenolics, with two aromatic (A and B) rings associated via C-C bonds by a 3 C oxygenated heterocycle. On the basis of the oxidation state of the central ring, flavonoids are further divided into anthocyanins, flavonols, flavanols, flavones, flavanones and isoflavonoids. Carob fruits are particularly rich in flavonols such as quercetin, myricetin, kaempferol and their glucosidic derivatives. Quercetin and myricetin rhamnosides are usually the most abundant flavonoids in carob. The presence of flavones (apigenin, luteolin and chrysoeriol), flavanones (naringenin) or isoflavones (genistein) are of low abundance [53,61,64].

Tannins comprise the most characteristic group of polyphenols in carob fruits and contribute to their astringency. In carob juice the concentration of tannins is ten-fold higher than in grape juice and it is decreased with the progress of fruit ripening [65]. Tannins are classified into hydrolysable and condensed (or non-hydrolysable) forms. In general, hydrolysable tannins are considered as multiple esters of gallic or ellagic acid with glucose and products of their oxidative reactions and are known as galloyl tannins and ellagitannins, respectively [52]. On the other hand, condensed tannins are non-hydrolysable oligomeric and polymeric proanthocyanidins [66]. Avallone and co-workers reported the presence of hydrolysable and condensed tannins in different parts of carob fruit [67]. In particular, carob pods contain a mean value of 2.75 mg condensed tannins/g and 0.95 mg hydrolysable tannins/g. Germ comprises higher concentration of tannins (16.2 mg condensed tannins/g and 2.98 mg hydrolysable tannins/g), whereas their concentration in carob seeds exists in traces. From a chemical point of view, carob tannins are mainly condensed tannins (proanthocyanidins), composed of flavan-3-ol groups and their galloyl esters, gallic acid, (+)-catechin, (−)-epicatechin gallate, (−)-epigallocatechin gallate, delphinidin, pelargonidin and cyanidin [53,61].

### 2.6. Amino Acids

The amino acid content of carob fruits consists of a mixture of 17 residues (aspartic acid, glutamic acid, serine, glycine, histidine, arginine, threonine, alanine, tyrosine, valine, proline, methionine, isoleucine, leucine, cysteine, phenylalanine and lysine) [36,68]. Aspartic acid, asparagine, alanine, glutamic acid, leucine and valine together comprise ca. 57% of the total amino acid content of the pods [63]. In general, carobs can be considered a good source of amino acids according to World Health Organization (WHO) standards for protein. More specifically, it contains all seven essential amino acids (threonine, methionine, valine, isoleucine, leucine, phenylalanine and lysine) at concentrations that meet the WHO standards [68].

### 2.7. Minerals

Carob fruits are an excellent reservoir of potassium and calcium. Potassium content ranges between 970 mg 100 g^−1^ dry weight and 1120 mg 100 g^−1^ dry weight, whereas the concentration of calcium reaches up to 300 mg 100 g^−1^ dry weight [36,63,68,69]. Taking into consideration that cow milk contains an average of 1200 mg calcium per liter, a portion of carob fruit contains an almost equivalent concentration of calcium with a cup of milk [70]. Macrominerals such as phosphorus and magnesium have been also found in carob fruits at lower concentrations. Carob fruits also contain many microminerals including iron, copper, zinc, manganese, nickel, barium, cobalt, etc. Among the microminerals, iron has the highest concentration. Finally, its seeds generally contain higher macro- and microminerals than the pods [36,63,68,69].

## 3. Health Benefits of Carob

Numerous studies have revealed several physiological responses to carob fruit and its products that may be relevant to the promotion of human health and the prevention or treatment of some chronic diseases. Below we categorize the health benefits of the carob fruit in cancer, diabetes, diarrhea, and hyperlipidemia. An emphasis on the clinical trials that carob has been subjected to is also provided. Table 2 highlights the chemical components of carob and their associated evaluation in human health.

### 3.1. Anti-Proliferative and Apoptotic Activity against Cancer Cells

Carob is rich in phytochemical compounds that have been shown in the literature to have antitumor, anti-proliferative and proapoptotic activity. For example, quercetin, a widely studied polyphenol, promotes apoptosis in T-leukemic cells by targeting directly the antiapoptotic protein Bcl-xL [76]. In addition, quercetin reduced the tumor size and inhibited angiogenesis in pancreatic and breast cancer xenograft models, indicating that it is also effective in the tumor microenvironment [77,78]. Gallic acid, a phenolic acid that exists in carob fruit, decreased the growth of osteosarcoma MNNG/HOS xenograft tumors in mice. When applied in the osteosarcoma cell lines MNNG/HOS and U-2OS, it caused apoptosis and inhibition of proliferation, while p-38 protein was found to be upregulated, JNK downregulated and ERK1/2 activated [79]. Finally, delphinidin, from the group of anthocyanidins, was found to affect many regulators of the NF-κB proteins expression and inhibited tumor growth in PC3 xenograft mice models [80]. Considering the above, it is a logical step to hypothesize that carob could act as a chemopreventive agent. Moreover, chewable tablets and fruit bars rich in carob fiber have been patented as chemopreventive agents [81].

Carob pods’ aqueous extracts have been evaluated for their anti-proliferative activity against hepatocellular carcinoma [82]. The authors explored the proliferation reduction of T1 mouse cell line caused by the two extracts via bromodeoxyuridine (BrdU) assay and estimated the half maximal inhibitory concentration (IC_50_) values between the range of 0.2–0.4 mg/mL. Additionally, after a 24 h treatment of the T1 cells with the extracts, they observed DNA fragmentation and activation of the caspase 3 pathway, showing that some components of carob can trigger apoptosis. High performance liquid chromatography analysis of the pod extracts demonstrated three main constituents: gallic acid, (−) epigallocatechin-3-gallate and (−) epicatechin-3-gallate.

The high presence of gallic acid in carob extract has been pointed out by Klenow et al*.* [32,83,84]. The authors hypothesized that the anti-proliferative effect in HT29 and LT97 human colon cancer cell lines may occur due to the high existence of gallic acid in its unconjugated form [32]. The carob fiber extract inhibited the proliferation of HT29 and LT97 cells by blocking DNA synthesis, but when gallic acid was applied alone to the cell lines, it did not exert the same effect [32]. These results indicate that the anti-proliferative activity of the carob extract is due to another constituent or it is an outcome of a synergistic effect of various components.

Custodio et al. evaluated the activity of carob germ flours’ methanolic extracts, from different genders and cultivars, in HeLa cervical cancer cells. Theophylline, a widely known alkaloid, was found to be the major component of the extracts. Testing the extracts in HeLa cells via WST-1 colorimetric assay, the proliferation was reduced with IC_50_ values varying between 2.7−10.3 mg/mL and was attributed to the existence of phenolic compounds and theophylline. The different anti-proliferative activity of each extract reveals its dependence from the phytochemical composition. The authors re-approached the same hypothesis by testing the anti-proliferative effects of carob fruit pulps in MDA-MB-231 breast cancer and HeLa cell lines [62]. The reduction of cancer cell viability occurred via apoptosis. Results also showed that the extracts had a concentration- and time-dependent effect on cell viability. Regarding the bioactive composition, catechin and gallic acid were the major phytochemicals in the methanolic extracts. The gender and the cultivar of the trees were shown, again, to play an important role in the phytochemical constitution. Specifically, the extracts that came from hermaphrodite trees displayed higher levels of phenolic compounds and thus higher anti-proliferative effect.

Overall, polar extracts of carob pods inhibit the proliferation of many cancer cell types. The induction of apoptosis seems to be the predominant mode of action for carob extracts. Although gallic acid is the most abundant phytochemical in polar extract, its cytotoxic effect is lower compared with carob extracts. The anti-proliferative activity of carob extracts could be correlated with synergistic effects among different components. Different genders and cultivars of carob affect the phytochemical profile and thus the anti-proliferative activity. Finally, further studies need to be conducted in order to clarify in which cancer type carob is more effective and sieve the exact mechanisms through which it acts upon cellular microenvironment. A deciding factor for its chemopreventive potential could be the in vivo experiments.

### 3.2. Anti-Diabetic Effects

The anti-diabetic effect of herbal preparations containing carob and other natural products has been evaluated. These preparations had low glycemic index when used as dietary supplements for people with diabetes [85].

Diet with carob gum was shown to decrease the glucose levels in rat blood [86]. In addition, Dos Santos et al. calculated the glycemic index of carob flour at 40.6 ± 0.05 via enzymatic hydrolysis in vitro [87].

The presence of d-pinitol in carob products could be responsible for the anti-diabetic effects as it regulates blood sugar level in patients with Type II diabetes mellitus by increasing insulin sensitivity [71]. Carob syrup is considered as a rich source of d-pinitol; as 10 g of it are sufficient, in comparison with the standard dose (10 mg d-pinitol/kg body weight), to lower the blood sugar level in type II diabetes [33]. Bates et al. proposed that d-pinitol could exhibit similar action with insulin and, thus improve glycemic control by testing its efficacy in animal models with diabetes [29]. The authors also presented that d-pinitol caused higher absorption of glucose in L6 muscle cell line, suggesting its implication in the glucose metabolic pathway in muscles rather than the greater production or the enhancement of action of insulin.

### 3.3. Anti-Diarrheal Effects

Carob and fractions of it are recommended to treat diarrhea symptoms. Previous study reported that a 2% solution of carob is able to block hemagglutination and adherence of *E. coli* on isolated intestinal epithelial cells. Blockage of adherence of bacteria isolated from the upper small intestinal tract of children could explain the effectiveness of proposed fraction [34].

Regarding bioactive composition, Loeb et al. explored the efficacy of carob pods’ tannins on the treatment of acute-onset diarrhea [88]. The fraction was composed by 40% tannins or 21.2% polyphenols and 26.4% dietary fiber. It is not the first time that tannins are considered responsible for anti-diarrheal action. Liu et al. proved through in vivo and in vitro techniques that the tannin extract of rhubarb downregulates the PKA/p-CREB pathway and consequently the expression of Aquaporins 2 and 3 which control the water transfer inside the cells [89]. Wursch (1991) also patented a dietetic product with anti-diarrheic activity that the particulated carob pod contains at least 20% by weight, based upon dry matter, of water-insoluble tannins [90].

### 3.4. Anti-Hyperlipidemia Effects

High levels of lipids or lipoproteins in blood may lead to atherosclerosis and afterwards to heart and vascular diseases. Therefore, supplements that reduce the lipid and cholesterol levels in blood are necessary to balance a high-fat diet. A cholesterol-reducing preparation comprising at least one dietary fiber selected from the group consisting of carob fruit flesh has been patented [91]. In addition, carob fiber in combination with n-3 fatty acids are the main ingredients of a patented foodstuff for positively influencing cardiovascular health [92].

The impact of carob components in hyperlipidemia has been studied thoroughly on in vitro and in vivo models. LBG was shown to reduce cholesterol and lipid levels in liver of rats fed a high-cholesterol diet by 10% [93]. However, in a more recent study, LBG did not exert an important effect on the cholesterol and triaglycerol levels of diabetic rats [94].

The studies concerning the carob fruit are less contradictory. When carob powder was administered to Sprague-Dawley rats along with a hyperlipidemic diet, cholesterol and triglycerides were lessened in a dose-dependent manner [95]. The authors also noted that the histopathological profile of the animal’s heart and kidneys remained normal in those that consumed the carob powder, whereas the rats that followed exclusively the hyperlipidemic diet presented severe abnormalities. Carob powder could be a potential candidate in diet regimen of obese and overweight persons. Valero-Munoz et al. observed that carob pod fibers suppressed physiological events that led to atherosclerosis in rabbits with dyslipidemia [96]. They observed that the expression of SIRT1 and PGC-1a, proteins with a key role in the vascular and metabolic system, were enhanced, proposing a new mechanism of action of carob components.

### 3.5. Carob and Clinical Trials

Clinical trials related to carob appear to use mixtures that contain carob components along with other substances [97,98,99,100]. The trials that refer exclusively to carob components associate mostly with infant regurgitation, hypercholesterolemia and diarrhea. This could be attributed to the limited number of studies concerning other diseases, such as cancer and diabetes. Treatment results about conditions like hypercholesterolemia and diarrhea can be discerned more easily and evidence of potency can occur in a shorter period of time.

#### 3.5.1. Infant Regurgitation

Vandenplas et al. tested and compared the efficacy of two ant-iregurgitation formulas (ARF) for the treatment of infant regurgitation [7]. Both ARF-1 and ARF-2 contained LBG and a double-blind cross-over trial was performed for one month in two groups of infants. The total number of infants was 115 with a mean age of 9.1 weeks for both groups and similar anthropometric parameters. ARF-2 was statistically more effective than ARF-1 with mean number of episodes of regurgitation decreasing from 8.25 to 2.32 for ARF-1 and to 1.89 for ARF-2. Same effect was observed for the mean score of regurgitation volume with ARF-2 presenting stronger action. Both ARF-1 and ARF-2 demonstrated efficacy in decreasing the volume and number of regurgitations in infants, offering a wider range of therapeutic options.

Miyazawa et al. examined the effects of two milk-based formulas containing LBG at different concentrations in infants with regurgitation episodes [6]. In this study, 39 infants with more than three episodes of regurgitation per day were examined. The comparison in gastric emptying of the two milk-based formulas was evaluated after feeding at certain time points. The first formula contained LBG at 0.35 g 100 mL^−1^ (HL-350) while the second 0.45 g 100 mL^−1^ (HL-450). On the contrary, regular formula (HL-00) was free of LBG. The evaluation of their effect on regurgitation episodes was tested on 27 infants with episodes that were randomly assigned to receive the formulas along with HL-00 for one week. Results showed that regurgitation episodes were significantly lower for infants that were fed with HL-350 or HL-450 rather than with HL-00. The comparison of the two formulas showed that HL-450 presented a slower rate of gastric emptying in infants with gastro-esophageal reflux.

Miyazawa et al. on previous studies examined the effects of two milk-based formulas containing LBG at different concentrations in infants with regurgitation episodes [5]. Formula HL-350 demonstrated promising potential by reducing regurgitation episodes in four-month-old infants with reflux. In this study, researchers examined the effect of HL-350 in infants less than two months. For this purpose, 20 infants with more than three episodes of regurgitation per day were assigned on a two-week study. The infants were separated into two groups, and the first group was fed HL-350 the first week and control milk (HL-00) the second. The reverse order was followed by the second group. Significant reduction in the number of regurgitation episodes was observed in infants during the week they were fed HL-350 in comparison to the week with control milk. Moreover, HL-350 did not affect the gastric emptying delay.

LBG was successfully administered for the treatment of gastroesophageal reflux in infants [8]. Twenty full term Thai infants with mean age 13.4 ± 7 weeks were assigned for this purpose to a four-week study. The clinical trial showed that the addition of CBG in milk formula improves the clinical symptoms of regurgitating infants but does not significantly affect the gastric emptying physiology.

#### 3.5.2. Hypercholesterolemia

In another study, Ruiz-Roso et al. examined the beneficial effect of a concentrated polyphenols extract from carob, with a high ratio of polyphenols, on serum lipids in humans [74]. In this double-blind clinical study, 88 volunteers with hypercholesterolemia (200–299 mg/dL) were divided into two groups, consuming placebo or fiber with insoluble polyphenols twice a day for four weeks. Triglycerides and low-density lipoprotein (LDL) and high-density lipoprotein (HDL) cholesterols were assessed at the start and after four weeks. According to the authors, total cholesterol, LDL cholesterol, LDL: HDL cholesterol ratio and triglycerides were reduced by 17.8% ± 6.1%, 22.5% ± 8.9%, 26.2% ± 14.3% and 16.3% ± 23.4%, respectively. On the other hand, the placebo group did not present any significant differences on their lipid profile. As the authors state, beneficial effects on human lipid profile can be seen with the consumption of fiber rich in insoluble polyphenols and this can help control hyperlipidemia.

In order to study the effect of LBG in familiar hypercholesterolemia, Zavoral et al. employed two groups, both containing adults and children [101]. Among the subjects, 18 presented familial hypercholesterolemia and 10 were normal. LBG was administered through food consumption to group A, while group B consumed food without it. The results showed that HDL was increased in both groups, but LDL and cholesterol were decreased in children with familial hypercholesterolemia. The cholesterol and LDL levels decreased more in the subjects with familiar hypercholesterolemia and, generally, LBG exhibited greater effect on the diseased adults rather than the children. However, this trial used a small number of subjects and the conclusions must be further validated.

Another study focusing on the benefits of carob pulp on serum cholesterol of hypercholesterolemic patients was conducted by Zunft et al. [31]. A total of 58 volunteers with hypercholesterolemia were assigned to participate in a double-blind, placebo-controlled clinical study in order to investigate if high concentration of insoluble fiber contained in carob pulp has a beneficial effect on serum cholesterol. Total, LDL and HDL cholesterol along with triglycerides were examined at the start of the study and after week 4 and 6 for every participant. The daily lunch schedule contained bread and a fruitbar along with 15 g/day of a carob pulp preparation or without. According to the results, LDL cholesterol was reduced by 10.5% ± 2.2% as was the level of triglycerides in females by 11.3% ± 4.5%. Additionally, the LDL:HDL cholesterol ratio was diminished by 7.9% ± 2.2% in the carob fiber group in comparison with the placebo group. The authors state that females presented better lipid profile at the end of six weeks than those of males.

#### 3.5.3. Diarrhea

Comparison of two different treatments of acute diarrhea, one containing carob bean juice (CBJ) combined with standard oral rehydration solution (ORS) and the other just ORS, was conducted by Akşit et al. in 80 children that were admitted to hospital with acute diarrhea and dehydration [75]. The subjects were randomly assigned to receive treatment with ORS, in accordance with WHO guidelines, and a combination of CBJ and ORS. Results showed that duration of diarrhea was reduced by 45% if children received ORS and CBJ together, also reducing the requirement of ORS by 38% in comparison with the children that received ORS alone. The researchers thus concluded that CBJ could play a significant role in the treatment of children’s diarrhea.

In another study, the water-insoluble carob fraction was successfully used in the treatment of diarrhea in infants aged 3 to 21 months [88]. The consumption of carob fraction improved bowel movements, body temperature and body weight after 24–48 h. According to the authors, the efficacy of the carob fraction was linked with its bactericidal effect on enteropathogenic *E. coli* and rotavirus.

The effect of insoluble carob fraction on traveler’s diarrhea was also determined by a double-blind, computer-randomized, placebo-controlled study of 755 volunteers [102]. The duration of the study was 48 h and results showed a positive trend for carob fraction but it was not efficacious in treating traveler’s diarrhea symptoms.

## 4. Carob’s Polyphenols and Bioavailability

Carob fruit is rich in polyphenols with phenolic acids, gallotannins and flavonoids being the most prevalent [103]. Although polyphenols illustrate a wide range of biological activities in vitro, their low bioavailability attenuates their efficacy in vivo [104,105]. Despite the fact that they are the most common in human diet, their activity is not always in parallel with their abundance, either because of poor absorption or rapid elimination [106]. Polyphenols present oral bioavailability of 10% (assays in animals) with a range around 2%–20%. A way to increase their bioavailability is of utmost importance since a large sample of the population would be needed for the clinical trials in order to demonstrate efficacy, and these assays would therefore not be affordable [107].

The first approach should be the study of the bioavailability and their pharmacokinetic profile since these are the main factors that affect their action, an aspect that is not thoroughly examined in most cases. Many potentially therapeutic phytochemicals present strong in vitro profile but diminished in vivo, as a result of their low ADME (**A**bsorption, **D**istribution, **M**etabolism and **E**xcretion) properties and limited cellular permeability. The basic parameters that can describe to a sufficient degree a pharmacokinetic profile are C_max_, T_max_, AUC and t_1/2_. C_max_ is the highest reached concentration of a drug after administration of one dose and before the administration of a second dose; T_max_ represents the time taken for the drug to reach the maximum concentration (C_max_); t_½_ is the time for the drug to reach half of its initial concentration; and AUC (area under the curve) refers to the total drug concentration over time [108]. The low absorption and intensive metabolism of the compounds with low bioavailability necessitate a more in-depth exploration of their bioavailability in animal and human studies [109]. A great number of polyphenols such as (−)-epiafzelechin, avicularin and isorhamnetin-3-*O*-neohesperidoside (flavonol 3-*O*-glycosides) have been determined using several liquid chromatography coupled with tandem mass spectrometry (LC-MS/MS) methods [110,111,112]. The majority of these studies to quantify flavonoids on in vivo models has been based on LC-MS/MS platforms; nonetheless, more research is needed in this direction [105,113].

The appraisal of their stability in human blood plasma with novel pharmacokinetic methods should be the basis upon which scientists should build in order to surpass the obstacle of low bioavailability. Further study of molecules with promising results should be undertaken in in vivo experiments since the gap between these two circumstances is sometimes unbridgeable.

## 5. LBG as Carrier Agent for Controlled Release of Drugs

In addition to its direct benefits to human health, LBG possesses a distinct role in the drug-delivery field as a carrier molecule. The galactomannan polysaccharide is composed of galactose and mannose monomers at a ratio of 1:4 and, because of its polymer properties, it can formulate particles by itself or synergistically with other polymers (Table 3) [114]. Furthermore, LBG has desirable physicochemical properties and other advantages, including: (i) it is biocompatible, bioresorbable and biodegradable in nature; (ii) it is a non-teratogenic and non-mutagenic food additive; (iii) it has an acceptable shelf-life; and (iv) its degradation products are excreted readily [115].

A potential polymer particle has to fulfill some crucial criteria for a successful, controlled delivery. The most important are the drug-loading efficiency, the slow release of the drug, the ability to penetrate and surpass biological barriers and the targeted delivery to the tissue of interest [116].

Dionisio et al. depicted in detail the applications of LBG in drug delivery [117]. Here, we present the new outcomes about formulations involving LBG solely or in combination with other polymers.

Maiti et al. composed beads of a carboxymethyl derivative of LBG and loaded them with Glipizide, a drug that is used for the treatment of type 2 diabetes [9]. The drug was loaded efficiently into the beads at a range of 93.35%–94.80% and found to be quite stable. When the complex was administered to rats, it caused a hypoglycemic effect that was maintained for 1–9 h. This could be attributed to the slow and controlled release of Glipizide from the beads. Another formulation with Ziprasidone HCl, an antipsychotic drug, has been also reported, whereby LBG was shown to provide high floatability when tablets were diluted in gastric fluids [10]. Panghal et al. managed to increase the solubility of Atorvastatin by loading it in a modified locust bean gum (MLBG), resulting in increased bioavailability of the drug in rats [11].

Other works developed mixtures of LBG with other polymers for greater functionality. Natural gums may display some flaws, like dysregulated swelling and fast release of the drug [12]. Thus, Kaity et al. created interpenetrating polymer network microspheres (IPNs) of LBG and poly vinyl alcohol (PVA) in different ratios, crosslinked via glutaraldehyde [12]. The ratio of LBG:PVA was found to affect the degree of release. In a following publication, Kaity et al. loaded to the LBG-PVA microspheres a vasodilator drug named Buflomedil hydrochloride and checked its pharmacokinetic parameters [13]. The IPNs improved Buflomedil availability as T_max_ was doubled. However, it did not exert satisfied biodegradability due to the higher amount of PVA in the microspheres. Other studies refer to the delivery particle formation of LBG with other polysaccharides like chitosan, algin and xanthan gum [14,15,16]. Several patents also demonstrated the great potential of LBG and its derivatives as carrier agent for controlled release of drugs. For example, LBG is used as a thickening agent for the production of pharmaceutical foam carrier [118], and as a cohesive composite in a controlled release insufflation carrier [119].

In conclusion, the main interest has turned mostly to use of LBG in combination with other polymers because their synergy helps counterbalance each other’s disadvantages. The ratio of the polymers plays a critical role in the degree of drug entrapment, as well as its stability and release rate. Regarding the aforementioned promising results, further investigation must be carried out so as to improve their delivery ability.

## 6. Economic Impact

The cultivation of carob tree is of great importance for Cyprus Island for environmental and economic reasons. Regarding the environment, the carob tree is considered a key crop for the long-term conservation of its high nature farmlands. On the other hand, carob production contributes to the local economy, albeit with decreasing contribution. Over the past decades, carob has been called the “black gold of Cyprus” due to its financial significance; it was the principal agricultural export. At present, carob cultivation on Cyprus has decreased dramatically as it is no longer considered profitable. Carob fruit is mainly used for the production of CBG from endosperm for the food industry—specifically for the production of carob syrup by extracting only water-soluble sugars—and, alternatively, as animal feed. Taking into consideration the health effects of carob, only the exploitation of carob fruit by the pharmaceutical industry would be able to provide viability to cultivation. The fibers and polyphenols originating from carob are currently not being used, although pharmacological studies have linked them with significant health-promoting properties. Thus, these constituents can become ingredients of novel functional foods, nutrition supplements and drugs of plant origin. Furthermore, research is needed to exploit LBG as a carrier agent for drugs and d-pinitol as a pharmaceutical preparation to treat diabetes. Overall, the exploitation of these pharmaceutical applications is expected to produce high added value products originating from carob fruit.

## 7. Data Collection

Our research included a thorough search of published literature in order to collect a significant amount of information related to carob and its properties. The main keywords we used were carob, composition, pulp, kibble, seed, health, biological activity, cancer, phenolics, locust bean gum, pinitol, amino acids, sugars, fiber, minerals, cancer, diabetes, hyperlipidemia, clinical trials. The databases searched were Scopus, Pubmed and Web of Science.

## 8. Future Directions—Conclusions

The research community, as well as the drug and food industries, have focused the last few years on the full exploitation of natural products—especially on phytochemicals—for the discovery of new pharmaceutical targets with promising potential and also for the enrichment of various food products with high biological-value natural substances. After thorough examination of the literature, it can be concluded that significant progress has been achieved in the field of natural products with their incorporation into new methods and protocols for drug discovery and food development, and that their manipulation has undoubtedly enormous potential.

Carob is a natural product that has drawn the attention of the research community not only because of its benefits to human health but also because of its significance to the economy and the environment. Overall, carob fruit contains many constituents of pharmaceutical interest. Indeed, carob bean gum (CBG) has mainly attracted scientific interest as it is widely used in the food industry and for pediatric purposes. Furthermore, carob fruit is an excellent source of d-pinitol and fibers, and some patents have already been registered for their recovery from carob fruit. Carob also contains a panel of polyphenols; gallic acid and its derivatives, as well as condensed tannins are the most representative phenolic compounds in carob fruit. The presence of some minerals and amino acids are also important for human health, so its implementation in a daily diet can help reduce incidents such as heart diseases, digestive disorders and diabetes in humans.

As far as human health is concerned, the carob tree and its fractions are associated with the prevention and treatment of a wide variety of diseases, including diabetes, hyperlipidemia, irritable bowel syndrome, and colon cancer. Numerous clinical trials have been conducted by researchers in order to explore the exact effects of carob fruit on humans. The results are quite encouraging, though a lot more effort should be invested in clinical studies to shed some light on the components and the molecular mechanism behind the actions of carob fruit in the human body.

For the better exploitation of natural products, new tools are necessary for the surveying of their bioavailability. New procedures are required that will amplify their biological profile, such as modification of carob in terms of encapsulation with calixarenes and cyclodextrins, as well as further evaluation of bioavailability and plasma stability profiles. Especially calixarenes possess structural features considered to be potential drug carriers (Drug Delivery Systems, DDS) due to their unlimited chemical modifications and their selective creation of complexes with biomolecules [120,121,122]. Carob fruit phytochemicals could be a potential candidate for formulations with calixarenes and cyclodextrins as a result of its extensive use in drug-delivery witnesses [9,11,117].

An integral part of these scientific procedures is state-of-the-art LC-MS/MS instrumentation that has flourished over the last few years. Their extended use for the determination of various compounds in extremely complex matrices makes them the ultimate tool in the field of pharmacokinetic studies [113]. Such a holistic approach could shape the base for the better exploitation of natural products in the drug and food industry, for the production of effective drugs and modified food products and for the confrontation of life-threatening diseases.

Over the last two decades, great development in different fields of life sciences has been performed due to the development of high-throughput omics technologies. High-throughput omics technologies are based on state-of-the-art analytical methods and allow a large number of measurements in a fairly short time period. Taking into consideration that carob fruit is a highly complex mixture of bioactive compounds and nutrients, the utilization of suitable omics technologies will promote research about the health-promoting properties of carob fruits and its by-products. Metabolomics, foodomics and phytochemomics are interesting approaches to this topic.

Metabolomics will provide an essentially unbiased, comprehensive qualitative and quantitative overview of the metabolites present in carob fruit as current studies are focused on a group of bioactive compounds e.g., polyphenols, galactomannans, etc. Foodomics and phytochemomics technologies are able to make connections between food components, food, diet, health, and disease. These approaches will be useful to correlate the consumption of carob or carob constituents with specific health claims since a cause−effect relationship may be established between phytochemicals and health-promoting properties. Furthermore, they will facilitate study of the effect of carob storage and processing on its bioactivity. It is also expected to be useful for optimizing the formulation of innovative functional foods, medicinal preparations and food supplements.

## Figures and Tables

**Table 1:** A summary of most common phenolic compounds in different parts of carob fruit.

Polyphenol	Carob Part/Fraction	Reference
**Phenolic acids**		
4-hydroxybenzoic acid	Pulp	[57]
Caffeic acid	Pulp	[57]
Chlorogenic acid	Seed	[60]
Cinnamic acid	Fiber, pulp	[57,61]
Coumaric acid	Fiber, pulp	[57,61]
Ferulic acid	Fiber, pulp, seed	[57,60,61]
Gallic acid	Pulp, fiber, seed	[53,60,62]
Gentisic acid	Seed	[60]
Syringic acid	Pulp, seed	[57,60]
**Flavonoids**		
(epi)gallocatechin	Fiber	[53]
(epi)gallocatechingallate	Fiber	[53]
Apigenin	Fiber, pulp	[57,61]
Catechin	Pulp, seed	[53,60,62]
Chrysoeriol	Fiber, pulp	[57,61]
Eridictyol	Pulp	[57]
Genistein	Pulp	[57]
Isorhamnetin	Fiber, pulp	[57,61]
Kaempferol	Fiber, pulp	[53,57,61]
Kaempferol rhamnoside	Fiber	[61]
Kaempferol-desoxyhexoside and -dihexoside	Pulp	[53]
Luteolin	Fiber, pulp	[57,61]
Myricetin	Seed	[60]
Myricetin rhamnoside and -desoxyhexoside	Fiber	[61]
Myricetin-hexoside	Fiber, pulp	[53,57,61]
Naringenin	Fiber, pulp	[57,61]
Quercetin	Fiber, seed	[60,61]
Quercetin-arabinoside	Fiber	[61]
Quercetin-desoxyhexoside and -hexoside	Fiber, pulp	[53]
Quercetin rhamnoside	Pulp	[57]
Tricetin 3′, 5′ dimethyl ether	Fiber, pulp	[57,61]
**Tannins**		
(epi)gallocatechin + 4 gallic acid units	Fiber	[53]
Hexose + 2 or 3 or 4 or 5 Gallic acid Units	Fiber	[53,61]
Pentoses + 2 gallic acid units	Fiber	[53]
prodelphinidin dimer and trimer	Fiber	[53]

**Table 2:** The chemical components of carob and their biological evaluation

Group of Chemical Constituents/Individual Substances	Biological Evaluation of Constituents/Disease	Carob Parts/Fraction	Reference
LBG/galactomannan	Gastrointestinal effects	Seed endosperm	[5,6,7,8]
d-Pinitol	Anti-diabetic activity	Carob pulp	[29,71]
Soluble and Insoluble Dietary Fiber Polyphenols/Gallic acid, Gallotannins, Flavonol Glycosides	Glycemic control, Enhanced lipid metabolism, Lowers total and LDL cholesterol	Carob Pulp	[30,31]
Insoluble Dietary Fiber Polyphenols/Tannins, Cellulose, Semicellulose, Lignin, Pectin	Cholesterol metabolism, Enhances lipid oxidation, Lowers postprandial acylated ghrelin	Carob Fiber	[72,73]
Polyphenols/ Gallic acid, Catechin, Myricetin rhamnoside, Eriodictyol glucoside, Quercetin glucoside, Quercetin rhamnoside	Anticancer effects	Carob Fiber	[32]
Polyphenols—Alkaloids/(+)-Catechin; Gentisic acid; Chlorogenic acid; Catechol; Ferulic acid; Gallic acid; Myricetin; Methyl gallate; Quercetin; Rutin; Syringic acid; Theophylline; Vanillin	Cytotoxic activities	Germ Flour Extracts (seed)	[31]
Fiber	Nutritional utilization, Induction of lipodemia	Carob Fiber	[33]
Fiber	Hyperlipidemia effects	Carob fiber	[31,74]
Tannins—Polyphenols	Anti-diarrheal effects	Carob pod	[34]
Tannins—Pectin	Anti-diarrheal effects	Carob bean juice	[75]

**Table 3:** Representative applications of locust bean gum (LBG) as drug carrier in drug formulations.

Carrier Agent	Role of Carrier Agent	Active Substance	Reference
Carboxymethyl derivative of LBG	Controlled release	Glipizide	[9]
LBG	Controlled release, Floatability	Ziprasidone HCl	[10]
Modified LBC (heating method)	Increased solubility	Atorvastatin	[11]
Interpenetrating polymer network microspheres of LBG and poly (vinyl alcohol)	Oral controlled release	Buflomedil HCl	[13]
LBG and chitosan mixtures	Mucoadhesive component in tablets	Propranolol HCl	[14]
Ca^2+^ alginate–LBG microspheres	Prolonged release	Aceclofenac	[15]
LBG gel-embedded niosomes	Preparation of vesicular systems	Monoammonium glycyrrhizinate	[16]

## Abbreviations

LBG	locust bean gum
CBG	carob bean gum
d.m.	dry matter
IC50	half maximal inhibitory concentration
BrdU	bromodeoxyuridine
AGE	advanced glycation end product
ARF	anti-regurgitation formulae
LDL	low-density lipoprotein
HDL	high-density lipoprotein
CBJ	carob bean juice
WHO	World Health Organization
ORS	oral rehydration solution
IPN	interpenetrating polymer network
MLBG	modified locust bean gum
PVA	poly (vinyl alcohol)
T_max_	time that drug is present at the maximum concentration in serum
LC-MS/MS	liquid chromatography tandem mass spectrometry
